# Innovations in communication training for medical and nursing students: Virtual reality communication tool for application and evaluation with key stakeholders and students (VR-TALKS) – a study protocol^☆^

**DOI:** 10.1016/j.pecinn.2025.100426

**Published:** 2025-08-26

**Authors:** Hanna A.A. Röwer, Aleksandrina Skvortsova, Mohamad M. Saab, Irene Hartigan, Claudia Bausewein, Sandra Martins Pereira, Pablo Hernández-Marrero, Jan Hrdlička, Kateřina Rusinová, Martin Loučka, Lucie Hrdličková, Martin Zielina, Cathy Payne, Liesbeth M. Van Vliet, Malte Klemmt, Kambiz Afshar, Stephanie Stiel

**Affiliations:** a Institute for General Practice and Palliative Care, Hannover Medical School, Germany; b Leiden University, Department of Health and Medical Psychology, the Netherlands; c Catherine McAuley School of Nursing and Midwifery, University College Cork, Ireland; d LMU University Hospital Munich, Ludwig-Maximilians-Universität München, Germany; e Universidade dos Açores | Fundação Gaspar Frutuoso, CEEAplA: Center of Applied Economic Studies of the Atlantic, Ponta Delgada, São Miguel Island, Azores, Portugal; f 3dsense, Czech Republic; g First Faculty of Medicine, Charles University, Prague, Czech Republic; h Department of Medical Psychology and Ethics, Masaryk University, Brno, Czech Republic; i Second Faculty of Medicine, Charles University, Prague, Czech Republic; j European Association for Palliative Care, Belgium

**Keywords:** Virtual reality, Healthcare education, Communication skills, Bad news delivery, Medical simulation, Medical education

## Abstract

**Background:**

In healthcare education, virtual reality (VR), simulating real-world situations, is emerging as a tool to improve communication skills, particularly in sensitive scenarios involving patients and caregivers. While promising, VR-based education also poses challenges such as avatar realism, cognitive load, and the need for pedagogical grounding.

**Objective:**

This protocol paper presents the VR-TALKS project, which aims to develop, apply, and evaluate VR scenarios designed to teach healthcare students communication skills in serious illness scenarios. Barriers and facilitators to integrating VR into healthcare teaching modules, along with the usability, feasibility, and educational impact of the VR tool, will be assessed across five European countries, incorporating insights from both students and educators.

**Methods:**

Phase 1 involves screening current communication courses at six partner institutions to identify opportunities for integrating VR. Phase 2 assesses the barriers and facilitators faced by approximately *n* = 70 educators in incorporating VR into communication training. Phase 3 focuses on developing VR scenarios based on the SPIKES and NURSE techniques. Phase 4 evaluates the usability and feasibility of the scenarios with *n* = 200 students and *n* = 30 educators. Feedback from this phase will inform further improvement of the tool.

**Expected results:**

The project will provide valuable insights into the barriers and facilitators of VR integration, develop two VR scenarios in multiple languages, and collect data on feasibility, usability, and user satisfaction. Additionally, it will offer recommendations for effectively incorporating VR into university curricula. Potential limitations of immersive VR, such as motion sickness, will be considered during evaluation.

**Conclusions:**

The project aims to enhance teaching methods for serious illness communication across Europe. The knowledge gained will be disseminated publicly through peer-reviewed publications and the project website, with plans to offer the VR training to other universities.

**Innovation:**

By addressing the limitations of conventional training, VR-TALKS offers healthcare professionals the opportunity to develop crucial communication skills in a repeatable, standardized, and time-flexible environment.

**Funding:**

ERASMUS+ Program through the Centre for International Cooperation in Education in the Czech Republic, “Dům zahraniční spolupráce” (DZS), spanning from 01.09.2023 to 31.08.2025.

## Highlights

• Development of VR training tool for healthcare students on serious communication.
• Integration of the SPIKES and NURSE frameworks into VR scenarios.
• Identification of barriers and facilitators for incorporating VR in education.
• Evaluation across six European universities to assess usability and feasibility.
• Innovation in communication training, offering repeatable, and adaptable learning.

## Introduction

Virtual reality (VR) is a technology that creates an immersive, computer-generated environment in which users can interact with simulated surroundings in a seemingly real or physical way [1]. This experience is typically achieved using VR headsets and controllers, enabling users to see, hear, and sometimes feel the virtual world around them. VR is not only transforming fields like gaming and entertainment. It also has a significant impact on healthcare education [2].

A recent literature review emphasized the role of VR in healthcare education, particularly in enhancing non-technical skills such as communication and teamwork [3]. Furthermore, VR as an interactive learning environment has been shown to be an effective tool for training healthcare professionals in the delivery of serious illness communication [4]. A team from the United States using 360° videos and another team from Belgium using a virtual patient showed that VR can enhance communication skills among healthcare students, particularly in improving empathy and thus in understanding patients' perspectives [5,6]. In the VR context, such improvements can be explained by Proteus effects (users' behavior changes based on their avatar characteristics) [7] and Presence effects (the feeling of ‘being there’ in the virtual environment) [8]. Another study involving virtual patients further demonstrates that VR is not inferior to other teaching methods [9]. These findings suggest that VR has the potential to be a valuable tool in healthcare education, particularly in teaching how to communicate effectively about serious illness and difficult news. Despite these positive outcomes, there are challenges and negative consequences that need to be reported. Open questions concern, for example, the authenticity of avatars [10], potential increases in cognitive load [11], the need for strong pedagogical grounding [12], or infrastructural barriers [13]. Physical side effects such as cybersickness, headaches, and eye strain have also been reported [14], as well as educational and social risks like over-reliance on VR [15]. Nevertheless, research shows that VR training has advantages over regular training methods, such as being more engaging, cost-effective, and standardizable [[16], [17], [18]]. However, further research is required to optimize the design and integration of VR training in health education, to test it in new fields of application and to develop VR further.

This short communication outlines the study protocol of the project VR-TALKS (Virtual Reality Communication Tool for Application and Evaluation with Key Stakeholders and Students), which aims to develop VR scenarios for training healthcare students in the effective communication of difficult news to patients as well as their relatives and caregivers. Following its development, the usability, feasibility, and educational impact of the VR scenarios will be evaluated among students and educators at six universities across five European countries. Furthermore, the perceived acceptability, predictors of intention to use, facilitators and barriers, perceived advantages and disadvantages of integrating VR in healthcare serious illness communication training will be investigated.

## Project consortium

**Fig. 1:**
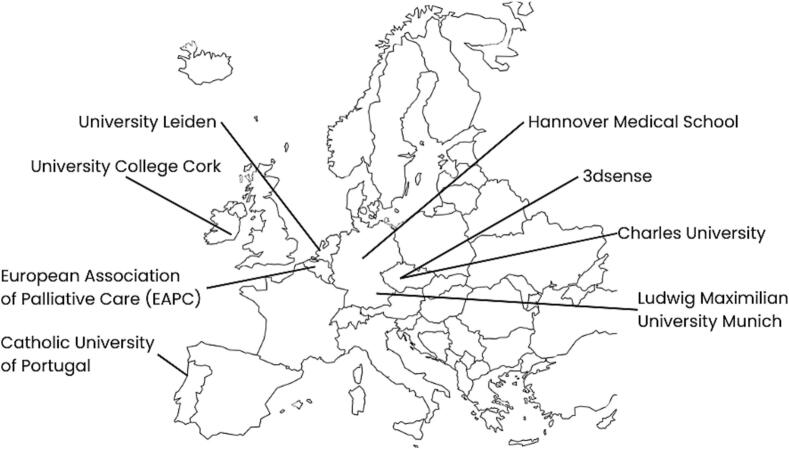
Project partners from Belgium, Czech Republic, Germany, Ireland, Netherlands, and Portugal.

The consortium consists of six European universities from Germany (two institutions), Portugal, Ireland, the Netherlands, and the Czech Republic. All are involved in the teaching of communication skills for healthcare professions students (e.g., medical and nursing students) and have experience in educational research. In addition, one European research organization contributes to the project (see Fig. 1). The consortium is led by 3dsense, based in Prague, which is active in the technical development of VR modules for teaching healthcare professionals. Together with its startup ComGuide, it has already been involved in the teaching of communication skills at Charles University in Prague.

## Project phases

**Fig. 2:**
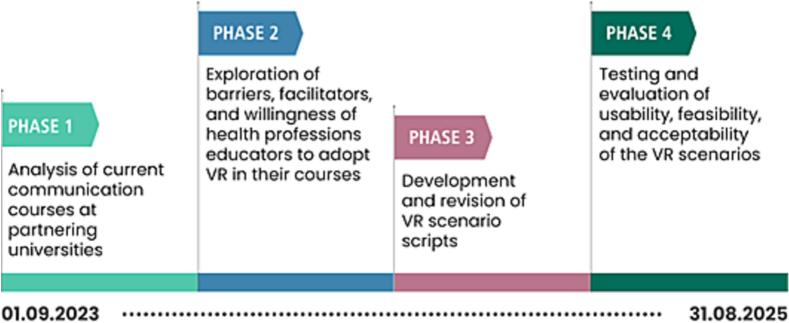
Phases and timeline of the project VR-TALKS.

The project consists of four consecutive phases (see Fig. 2) and will span over 24 months. Fig. 2 illustrates the phases and timeline of the project.

## Steps towards implementing and evaluating the new VR Tool

### Phase 1

In the first phase of the project, potential current courses across all partner universities are reviewed to identify opportunities for testing and integrating the VR communication tool. These courses are designed for medical and/or nursing students and focus on the topic of delivering bad news within various undergraduate and postgraduate modules. Each partner university compiles an initial list of relevant teaching courses, including, for example, “Patient-Oriented Communication,” “Palliative Care,” “Bioethics and Psychology,” and “Diagnostic Methods.” This overview serves as a basis for further discussion and refinement in Phase 3.

### Phase 2

In Phase 2, we examine: i) the acceptability, ii) the predictors of intention to use, iii) the barriers and facilitators, and iv) the perceived advantages and disadvantages of incorporating VR into training for communicating about serious illness and bad news, from the perspective of health professions educators in Europe. The focus on educators reflects their key role in the implementation of curricular innovations. Students are not included in this phase, as the aim is to explore the preconditions and perspectives of those responsible for course planning and integration prior to actual deployment. A survey is conducted among health professions educators, utilizing an adapted version of the *Assessing Determinants of Prospective Uptake of Virtual Reality* (ADOPT-VR) [19] questionnaire to assess acceptability and predictors of intention to use. The ADOPT-VR, based on the Decomposed Theory of Planned Behavior [20], examines 11 constructs that determine the acceptability of new VR technology: attitudes, perceived usefulness, ease of use, compatibility, social norms, peer influence, superior influence, perceived behavioral control, self-efficacy, facilitating conditions, and behavioral intention to use the technology. In addition, educators are asked to describe barriers and facilitators, as well as the potential advantages and disadvantages of integrating VR into their teaching practices.

The cross-sectional exploratory online survey is distributed using snowball sampling through the networks of participating partners and social media channels. Approximately 70 educators involved in developing or teaching communication courses for medical and/or nursing students are expected to participate in the survey. Data will be collected electronically via the survey platform, transferred in aggregate form to the analysis software, and subsequently checked for plausibility. Quantitative data will be analyzed using descriptive statistics and multiple regression analysis. Responses to open-ended questions will be subjected to qualitative categorization.

### Phase 3

In Phase 3, the scripts and virtual environment are developed for two VR scenarios, based on established communication frameworks. An on-site kick-off meeting with all project partners is held to discuss existing teaching modules within the healthcare faculties. During this meeting, an existing VR application featuring a scenario involving an angry patient is demonstrated. Based on this, two new VR scenarios are created for testing within the project: one built on the SPIKES framework (Setting, Patient's Perception, Invitation, Knowledge, Exploring/Empathy, Strategy/Summary) [21], and the other on the NURSE framework (Name, Understand, Respect, Support, Explore) [22]. The scenarios feature two characters: a patient named Mrs. Ibrahim and a caregiver named Mr. Steward (see Image 1).

(1) **SPIKES scenario (see** Table 1**)**: A clinician (the student) meets with a patient called Nora Ibrahim (the avatar), a 45-year-old woman with advanced breast cancer. During the consultation, Nora is informed that her condition has worsened, and that no curative treatment options remain beyond palliative care. Using the SPIKES framework [21], the clinician (the student) provides support as Nora processes the news. Potential outcomes may include Nora agreeing to discuss the situation with her husband or feeling overwhelmed and requesting time alone.

**Table 1:** SPIKES Scenario

Description of the place	Consultation room - invite Nora in
Description of the characters on the scene	• Nora • 45 years old • Woman • African descent • Married to Hussein • Mother of two kids • Overweight due to prolonged use of corticosteroids • Has alopecia (total hair loss) due to radiotherapy to the brain • Scenario in consultation room • Nora is on her own
Background story	Nora has advanced breast cancer. Her disease dates back over three years and she has undergone multiple lines of therapy. Recent investigations confirmed extensive and progressive disease burden. Nora is currently receiving radiotherapy to the brain due to brain metastasis. As a result, she has alopecia (total hair loss) and severe headaches. Nora is profoundly fatigued. Her prognosis is limited to weeks/few months. Nora coped with her illness by “being positive.” She actively researched her disease and all treatment options. She views “negativity” as a failing and despite her massive burden of disease, continues to express a desire for more treatment – “what have I got to lose?” You asked to see Nora in your clinic/consultation room to discuss the situation and to advise her that there are no further treatment options other than pain and symptom management (i.e., no more radiotherapy to the brain since the tumour is not responding). She is married to Hussein but prefers not to bring her husband to consultations since he is busy with work and the kids.
Possible outcomes	[bad ending] Nora is upset (not angry). She is no longer open for further communication. She tells you that she does not want to hear about it anymore and needs some space to think. [good ending] Nora thanks you for taking the time to explain her situation. She wishes to speak to her husband Hussein first and then she will follow up with you after a few days.
Learning outcomes using SPIKES:	• **S - Setting:** The scenario unfolds in a consultation room where the door remains ajar, allowing some noise from the waiting area. Nora forgets to close the door behind her and stands, prompting the clinician to kindly ask her to close the door and take a seat. • **P - Perception:** The clinician starts by asking how Nora is doing, to which she responds that she is very tired and her headaches won't go away, even with all the medications. The clinician then confirms that she is currently undergoing radiotherapy for her brain, and Nora acknowledges that she has been on it for a while. When asked if she knows why she is there, she expresses that she thinks they will discuss her treatment or any new options for her brain tumour. • **I - Invitation:** The clinician affirms this and checks if Nora is comfortable discussing the details. Nora enthusiastically agrees, expressing hope for good news. When asked if anyone is with her, she explains that her husband, Hussein, wanted to come but is busy with work and the kids, so she told him not to. The clinician expresses understanding of her situation. • **K - Knowledge:** The clinician gently explains that the news is not what they had hoped for, which confuses Nora. They reveal that the brain scan results show the tumour is not responding to the radiotherapy. This shocks Nora, who was not expecting such news. She recalls reading that radiotherapy is usually effective for brain tumours and expresses her confusion and frustration, wondering if she needs more radiotherapy or perhaps chemotherapy. The clinician acknowledges her feelings, emphasizing the disappointment of not achieving better treatment outcomes, and explains that the tumour has not responded to previous treatments. • **E - Emotions:** As Nora processes the information, the clinician recognizes her shock and confusion. They allow a moment of silence for her to absorb the news, maintaining a compassionate presence. Nora questions if she should just wait for her headaches to worsen and expresses concern about what to tell Hussein and the kids. The clinician gently explains that further radiotherapy could do more harm than good and reassures her that there are options available to help manage her headaches and keep her comfortable. To support Nora emotionally, the clinician emphasizes understanding of her situation, saying, “I can see this is a lot to take in, and it's completely natural to feel overwhelmed right now.” Nora then reflects on the information before the clinician asks if she has heard of palliative care. Initially shocked by the term, Nora associates it with a negative connotation, thinking it's where people go to die. The clinician clarifies that palliative care provides many benefits, including better management of headaches and support for her family. Nora acknowledges that this is a lot to absorb and thanks the clinician for the honesty. She expresses the need to talk to Hussein before making any decisions and asks if that is okay. • **S - Strategy and Summary:** The clinician reassures Nora that it is completely fine to discuss the situation with her husband. They agree to stop the radiotherapy for now and suggest she talk to Hussein about both the discussion and the option of palliative care. Nora feels satisfied with this plan and expresses her gratitude. The clinician then checks if she has any questions, and Nora replies that she is okay for now but will speak with her husband and call later in the week. The clinician wishes her well, assuring her they will talk soon.

(2) **NURSE scenario (see** Table 2**):** A clinician (the student) interacts with Mr. Steward (the avatar), a 75-year-old man who is distressed upon learning that his wife will remain hospitalized instead of being discharged. He expresses disappointment, particularly as their 55th wedding anniversary is approaching. The clinician (the student) uses the NURSE framework [22] to acknowledge Mr. Steward's emotions and explore possible solutions, such as enabling family visits or arranging video calls. Outcomes range from reaching a compromise to Mr. Steward becoming upset and requesting to speak with a supervisor. Further details are provided in Appendix 1.

**Table 2:** NURSE scenario.

Description of the place	A character is standing in a busy hall. The student should take the relative to a private room and ask to sit down.
Description of the characters on the scene	• Mr. Steward • 75 years old • Male • Married to Mrs. Steward, who is currently treated in the hospital
Background Story	Mr. Steward is the husband of a patient Mrs. Steward. His wife was due to be discharged from the hospital today, but unfortunately the latest examinations have shown that Mrs. Steward must remain in hospital for a few more days. The husband got the news directly from his wife in the hospital room, and now he enters the nurses' station and confronts you with the situation. Mr. Steward becomes very silent and disappointed. He is saying: “But no one told me about it”. Then Mr. Steward will say that this weekend it will be a 55-year anniversary for them, the family arranged a big celebration and relatives are coming from abroad. Mr. Steward looks desperate.
Possible outcomes	[bad ending] Mr. Steward gets very upset and asks to talk to the supervisor. [good ending] A compromise is found. The student is offering for a small group of family to visit Mrs. Steward and the rest of the family can join the celebration online.
Learning outcomes using NURSE	• **N - Name:** As Mr. Steward approaches the nurses' station, the clinician acknowledges his feelings immediately. They might say, “Mr. Steward, I can see that you're feeling quite upset and disappointed right now.” • **U - Understand:** Next, the clinician expresses understanding of his situation. They could say, “It must be really frustrating to hear this news, especially since you were expecting your wife to come home today. I understand how much this means to you.” • **R - Respect:** The clinician respects his feelings by validating his concerns. For example, they might say, “It's completely understandable that you feel this way. You've been looking forward to celebrating your 55th anniversary with your family, and this sudden change is really hard to accept.” • **S - Support:** The clinician offers support and assistance. They might say, “I want to help you through this situation. Let's see what we can do to make things a bit easier for you and your family during this time.” • **E - Explore:** Finally, the clinician explores potential solutions together with Mr. Steward. They could suggest, “Perhaps we can arrange for a small group of your family to visit Mrs. Steward in the hospital, so she can be part of the celebration. We could also set up a video call for the rest of the family who can't be here. How does that sound to you?”

Scenario requirements are developed collaboratively in a structured iterative process involving all project partners, including medical educators, communication experts, clinicians, and VR designers. Criteria are discussed in project team meetings and determined, based on their pedagogical relevance and feasibility. Cultural differences are addressed through regular input from all participating countries to ensure scenarios are broadly applicable and sensitive to context. For example, communication norms and expectations are discussed and harmonized during cross-site meetings.

As for the VR environment, the technical team works closely with the content experts to ensure clinical realism (e.g., hospital setting, spatial design). The script development process focuses on creating realistic interactions within a limited set of avatar responses. These scripts are integrated into a VR application to enrich student experience during later phases of testing. ComGuide, a startup under 3dsense and responsible for the VR conversion and application development, uses its in-house expertise to execute this task. Following script development, preliminary testing takes place across partner organizations. This iterative process results in the creation of two communication tree scripts, which are translated into the national languages of all project partners.

After finalizing and approving the scripts, ComGuide converts them into video scripts. After incorporating feedback, the finalized versions are translated into each participating country's language. Audio recordings are produced in collaboration with professional dubbing studios and the universities. These recordings are then integrated into the VR application for testing in the next phase. Using motion capture technology, ComGuide converts the scripts into a VR-compatible format. Additionally, an AI system is used to generate dynamic dialogue pathways based on students' spoken input. The AI enables real-time interaction, triggering the appropriate avatar response in the simulation.

It is important to note that the virtual environment and technical application are not developed from scratch as part of the project. Instead, 3dsense, acting as the consortium leader, provides its pre-existing application to the project free of charge and modifies it as needed to meet the project requirements.

### Phase 4

In Phase 4, the usability, feasibility, and acceptability of the training is tested and evaluated. Students and educators from selected healthcare programs of the participating universities are invited to participate in VR module testing and a posttest survey using purposive and snowball sampling. Data is collected from each university, targeting at least 30 undergraduate and postgraduate medical or nursing students (totaling approximately 180 students) and 5–10 educators per site (approximately 30–60 educators in total) who are involved in teaching communication skills. Participants will engage with one of the two developed VR scenarios.

Students will engage with the chosen scenario twice. During the first attempt, participants receive guidance through on-screen prompts within the scenario to direct their responses and suggest communication strategies. In the second run, they proceed self-directed and simulate a more realistic interaction, with no guidance provided. This two-step approach balances structured learning and realistic practice. In most locations, students are able to choose which scenario to engage with. However, at the two German partner sites, students are assigned to a specific scenario in advance: in Hannover, the SPIKES scenario is used; in Munich, the NURSE scenario is implemented.

Within the current project, the VR training is designed primarily as a solo experience where students interact individually with the virtual patient. No recordings of the student sessions are made, and no individual feedback via video review is planned. However, at the end of each session, a brief, structured debriefing is conducted by an instructor/educator to reflect on the experience and to provide immediate feedback.

During the initial testing phase, technical support is available from the local project team to assist students and educators as needed. Immediately after completing the VR training, participants complete a survey [23].

The survey comprises five parts:

(1) Sociodemographic Questionnaire: Includes 10 closed items for students and 11 for educators, covering factors such as gender, teaching experience, and prior experience with VR.
(2) System Usability Scale (SUS): Features 10 agreement-scale items (e.g. “I thought VR was easy to use.”).
(3) Feasibility Questionnaire: Contains 10 agreement-scale items (e.g. “The VR scenario is applicable to real life.”).
(4) Satisfaction Questionnaire: Includes 2 satisfaction-scale items (e.g. “Overall quality of the VR scenario.”).
(5) Open-Ended Questions: Comprises 3 questions inviting feedback on what participants enjoyed most, enjoyed least, and areas for improvement in the VR scenario.

The survey will be administered in English, although respondents are welcome to answer open-ended questions in their native language.

Data will be collected electronically via the survey platform, transferred in aggregate form to the analysis software, and subjected to plausibility checks. Quantitative data will be analyzed using descriptive and inferential statistics in R, while qualitative responses will be analyzed through qualitative content analysis. Evaluation results will be systematically reviewed by an interdisciplinary project team – including VR developers, communication experts, educators, and clinical partners – to identify areas for improvement. This feedback loop will guide iterative refinements of the VR tool to optimize its usability. The Strengthening the Reporting of Observational Studies in Epidemiology (STROBE) checklist will be used to guide the reporting of this part of the study.

## Conclusion

The primary outcome of the project centers on advancing communication training through innovative VR applications in healthcare education. Key achievements include:

• The development of immersive VR scenarios and scripts designed to teach the delivery of bad news in healthcare contexts, grounded in widely recognized communication frameworks (i.e., SPIKES and NURSE).
• Comprehensive testing to evaluate the feasibility and usability of these scenarios across multiple universities.
• Dissemination of findings through scientific publications, conference presentations, and targeted outreach to key stakeholders.

The project aims at establishing VR as an effective and valuable tool in healthcare education, supported by evidence of its feasibility and usability as well as acceptabilty and perceived benefits among students, educators, and stakeholders.

Innovation in this context extends beyond the adoption of advanced technology to the transformative role VR plays in reshaping the learning process for handling difficult and emotionally charged conversations. Traditional teaching methods, such as role-playing or classroom-based lectures, often face challenges including limited realism, dependence on skilled facilitators, psychological pressure to perform in front of a group, and students' reluctance to fully engage in emotionally intense scenarios. VR-TALKS helps bridge these gaps by offering an immersive, interactive, and realistic learning environment. This platform enables students to practice complex communication scenarios with virtual patient and caregiver avatars, fostering deeper engagement and skill development.

The integration of evidence-based frameworks, such as SPIKES and NURSE, ensures that the VR scenarios combine technological innovation with pedagogical rigor. By simulating lifelike environments and encounters, VR supports not only technical communication skills but also empathy and adaptability – key-competencies in conversations about serious illness. Moreover, VR offers scalability and standardization, allowing for consistent training of large student cohorts across various institutions.

The current VR environment focuses primarily on verbal communication, and students do not see their own hands or body, nor can they physically move or use gestures within the simulation. The design prioritizes practicing structured communication frameworks in a controlled setting. However, the importance of nonverbal communication and physical presence is well recognized, and will be part of the evaluation.

Evaluating the long-term impact of VR on communication skills in healthcare education remains a significant challenge. Addressing this requires proactive planning, active stakeholder engagement, and the implementation of robust evaluation strategies to maximize both project success and educational impact. To ensure the project's long-term sustainability, the project will continuously disseminate updates and findings across Europe through journals, conference presentations, and online channels, including the website www.vr-talks.com and social media. In addition, the final VR scenarios with revisions based on the evaluation feedback, will be publicly released on our website under the MIT license, making them freely available for use and adaptation by interested universities and other institutions.

The following is the supplementary data related to this article.

**Supplementary Fig. S1:**
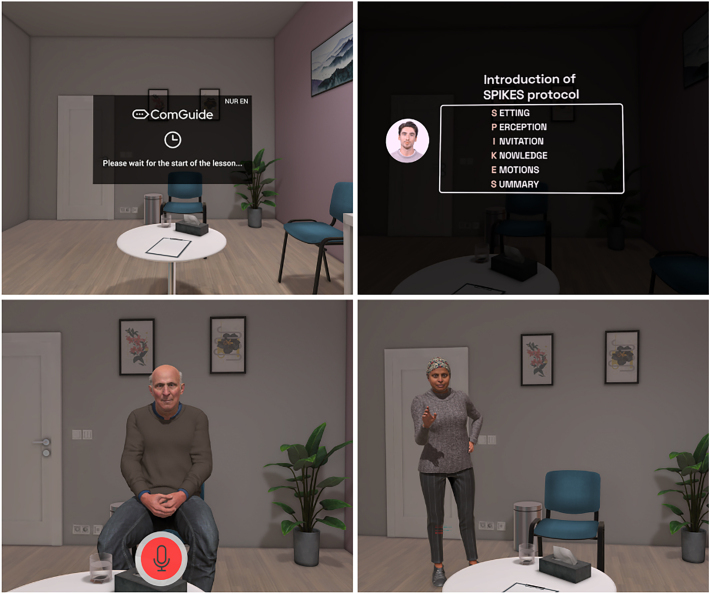
Screenshots of the VR application (top left: start of application; top right: introduction to the communication model SPIKES; bottom left: participant talks to Mr. Steward; bottom right: Mrs. Ibrahim talks to participant).

## CRediT authorship contribution statement

**Hanna A.A. Röwer:** Writing – original draft, Visualization, Project administration, Methodology. **Aleksandrina Skvortsova:** Writing – review & editing, Writing – original draft, Project administration, Methodology, Investigation, Formal analysis, Data curation, Conceptualization. **Mohamad M. Saab:** Writing – review & editing, Writing – original draft, Funding acquisition, Supervision, Resources, Project administration, Methodology, Investigation, Formal analysis, Data curation, Conceptualization. **Irene Hartigan:** Writing – review & editing, Funding acquisition, Resources, Project administration, Methodology, Investigation, Formal analysis, Data curation, Conceptualization. **Claudia Bausewein:** Writing – review & editing, Funding acquisition, Supervision, Project administration, Methodology, Investigation, Conceptualization. **Sandra Martins Pereira:** Writing – review & editing, Funding acquisition, Supervision, Project administration, Methodology, Investigation, Conceptualization. **Pablo Hernández-Marrero:** Funding acquisition, Supervision, Methodology, Investigation, Conceptualization. **Jan Hrdlička:** Writing – review & editing, Supervision, Software, Resources, Project administration, Methodology, Funding acquisition, Conceptualization. **Kateřina Rusinová:** Writing – review & editing, Funding acquisition, Supervision, Project administration, Methodology, Investigation, Conceptualization. **Martin Loučka:** Funding acquisition, Project administration, Methodology, Investigation, Conceptualization. **Lucie Hrdličková:** Writing – review & editing, Supervision, Project administration, Methodology, Investigation, Funding acquisition, Conceptualization. **Martin Zielina:** Funding acquisition, Project administration, Methodology, Investigation, Conceptualization. **Cathy Payne:** Writing – review & editing, Funding acquisition, Supervision, Project administration, Methodology, Conceptualization. **Liesbeth M. Van Vliet:** Writing – review & editing, Funding acquisition, Project administration, Methodology, Investigation, Formal analysis, Data curation, Conceptualization. **Malte Klemmt:** Writing – review & editing, Project administration, Investigation. **Kambiz Afshar:** Writing – review & editing, Funding acquisition, Supervision, Project administration, Methodology, Investigation, Conceptualization. **Stephanie Stiel:** Writing – review & editing, Supervision, Project administration, Methodology, Investigation, Funding acquisition, Conceptualization.

## Ethics

The study will undergo review by the research ethics committees of all participating institutions for approval. All participants will provide informed consent prior to using the VR application and completing the survey. Participation is entirely voluntary and may be withdrawn at any time without providing a reason. A distress protocol is put into place and will be activated if needed, e.g. if participants experience emotional discomfort during the study. Considering that the study is conducted in educational environments, all participants will be informed that they will not suffer any disadvantages if they refuse or discontinue participation in the survey or the testing. For students, neither participation nor nonparticipation will influence their evaluation in the module in which the testing is integrated. Confidentiality and anonymity are guaranteed to all the participants.

## Funding

The project is funded by the ERASMUS+ Programme through the Centre for International Cooperation in Education in the Czech Republic, “Dům zahraniční spolupráce” (DZS), spanning from 01.09.2023 to 31.08.2025 (24 months).

Sandra Martins Pereira was Principal Investigator funded by the Portuguese Foundation for Science and Technology (FCT) under the Scientific Employment Stimulus (CEECINST/00137/2018, DOI 10.54499/CEECINST/00137/2018/CP1520/CT0010) at CEGE: Research Centre in Management and Economics, Ethics and Sustainability Research Area - Palliative Care Research. This manuscript was partially written during the duration of this contract.

Sandra Martins Pereira and Pablo Hernández-Marrero would also like to acknowledge the financial support from FCT - Fundação para a Ciência e Tecnologia (Portugal) through the research grant UIDB/00685/2020 and UID/00685 of the Centre of Applied Economics Studies of the Atlantic (CEEAplA) - School of Business and Economics | University of the Azores and from the Regional Directorate for Science, Innovation and Development.

## Declaration of competing interest

The authors affiliated with 3dsense/ComGuide, the consortium leader, declare a potential conflict of interest due to the involvement of one member (JH) in the commercialization of VR products used in this project. Although this partner does not directly generate revenue from the VR application within this project, they are involved in marketing VR products employed in the research. Consequently, there is a possibility that project activities could indirectly benefit the partner's commercial interests by enhancing visibility, market perception, or future sales prospects for these VR products.

To ensure transparency and uphold scientific integrity, all project activities are conducted in accordance with the Declaration of Helsinki and the principles of Good Scientific Practice. Furthermore, the partner has committed to clearly separating their project role from their commercial activities and will refrain from influencing the project's execution or the content of resulting publications. 3dsense/ComGuide will not exert influence over decisions unrelated to the project's research objectives or the publication of research findings. All stakeholders will be regularly informed of any developments that might impact this disclosed conflict of interest, ensuring that the research remains objective and focused solely on its stated goals.

The Erasmus+ funding source had no role in the design of this study and will have no role in its execution, data analysis, and interpretation, or decision to submit or publish the results.
